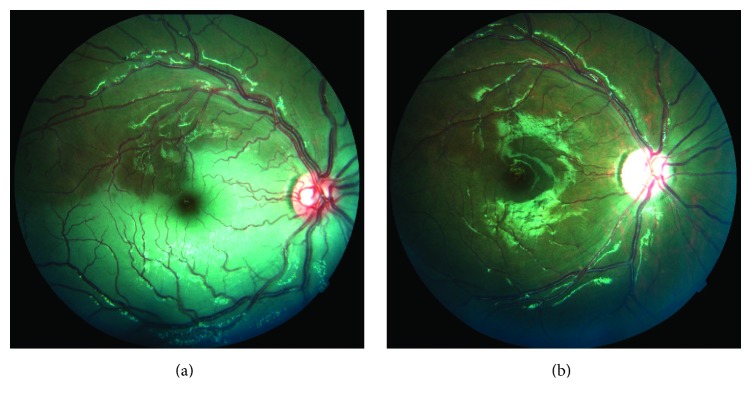# Hemiretinal Artery Occlusion in an 11-Year-Old Child with Dextrocardia

**DOI:** 10.1155/2016/5104789

**Published:** 2016-12-06

**Authors:** Diana E. Arévalo Simental, Enrique A. Roig Melo-Granados, Saúl Cortés Quezada, Manuel A. Páez Escamilla, Carmen L. Soria Orozco, Jorge E. Jacinto Buenrostro

**Affiliations:** Antiguo Hospital Civil de Guadalajara, Universidad de Guadalajara, Guadalajara, JAL, Mexico

## Abstract

*Purpose*. To report a case of hemiretinal artery occlusion in a child with dextrocardia, visceral heterotaxia, and secondary polycythemia.* Methods*. Complete clinical examination, fundus photography, and retinal fluorescein angiography were performed. Laboratory testing included complete blood cell count, homocysteine, protein c, protein s, activated protein s, methyltetrahydrofolate and homocysteine activator genes, factor leiden V gene, antithrombin III, and activated protein c resistance. In addition, transthoracic and transesophageal echocardiogram and cardiac catheterism were performed.* Results*. We report an 11-year-old boy with a sudden, painless visual loss in his right eye. His past medical history is remarkable for a congenital cardiac disease. He presented with vision of light perception in the right eye and a relative afferent pupillary defect. Fundus findings included a macular cherry-red spot and inferior hemiretinal whitening consistent with hemiretinal artery occlusion. Laboratory testing showed increased red blood cell (RBC) count, hemoglobin, and hematocrit. The patient was treated with four phlebotomies with improvement of RBC count and after one month reperfusion of the retina and a visual acuity of 20/200 were observed. Thrombophilia and cardiac screening were negative, except for secondary polycythemia.* Conclusion*. Hemiretinal artery occlusion is extremely rare in children and is often associated with congenital cardiac disease and hypercoagulative states.

## 1. Introduction

An 11-year-old boy presented to the ophthalmology department with a sudden, painless loss of vision of the right eye. His medical history is remarkable for a congenital cardiopathy and an ischemic stroke. Now he presents with hemiretinal artery occlusion.

## 2. Case Report

An 11-year-old boy presented with a history of sudden, painless visual loss of 24 hours. Family members denied headache or any associated neurologic complaints. The patient had a history of ischemic stroke at the age of 2 years and polycythemia secondary to a Glenn procedure performed to treat a left aortocaval juxtaposition, infundibular stenosis, and dextrocardia.

## 3. Systemic and Ocular Examination

**Figure 1:**
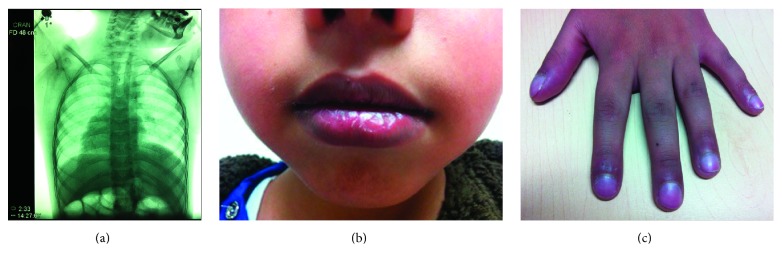


On systemic physical examination the patient had dextrocardia (Figure 1(a)), perioral cyanosis, (Figure 1(b)), drumstick fingers (Figure 1(c)), right hemiparesis, and normal sensibility.

On ocular examination of the right eye, the patient presented with a visual acuity of light perception and a relative afferent pupillary defect. Conjunctiva, corneal transparency, anterior chamber, and crystalline lens were normal. Intraocular pressure was 14 mm/Hg. On fundus examination, a whitening of the inferior hemiretina and a macular cherry-red spot were observed, along with a discrete increase in retinal vein dilation and a decreased retinal arterial size in the inferior arcades with apparent decreased perfusion of these vessels (Figure 2(a)). Fluorescein angiography showed an arm-retina time of 12 seconds; however, arteriovenous transit phase showed incomplete and delayed filling in the inferior arterial branch. Left eye exhibited no pathological changes with a 20/20 visual acuity.

A thrombophilia screening was performed that included homocysteine, protein C, protein S, activated protein S, methyltetrahydrofolate, homocysteine activator genes, factor leiden V gene, antithrombin III, activated protein C resistance, prothrombin mutation G20210A, lipoprotein A, antiphospholipid antibodies, and complement levels all which were negative. ESR, CRP, and liver and renal function were normal. Blood count reported increased RBC count, hemoglobin, and hematocrit (7.7 million cells/*μ*L, 19 mg/dL, and 60%, resp.). Cardiac screening consisted in a transthoracic echocardiogram and a cardiac catheterism, both reported a functional Glenn shunt. No ocular intervention was performed because of the time of presentation and the retinal and fluorescein angiography findings suggesting partial arterial occlusion in the inferior hemiretina. Hematology department decided to perform four phlebotomies in a 2-week period; RBC count, hemoglobin, and hematocrit after the treatment decreased to 5.5 million cells/*μ*L, 15 mg/dL, and 45%, respectively. Retinal arterial reperfusion and normal retinal color were observed (Figure 2(b)) two weeks following the occlusive event. A visual acuity of 20/200 was recorded after one month of follow-up. The patient was scheduled for a regular follow-up every 3 months with stable visual acuity.

## 4. Discussion

The average age in patients with retinal artery occlusions is 58.9 years [1]. The incidence of retinal artery occlusion in patients less than 30 years has been estimated to be less than 1 in 50,000 [1]. Retinal artery occlusion is an extremely rare condition in the pediatric population and most patients have some detectable risk factors. Cyanotic heart disease or hypercoagulable states are the most frequent conditions associated. In such cases, emboli originate from a heart valve defect, which include mitral valve stenosis, pulmonary valve stenosis, and left ventricular hypertrophy [2, 3]. Secondary polycythemia is a well-documented etiologic factor related to thrombotic events, both arterial and venous, as they include retinal artery occlusions [4]. The 2 major risk factors for developing vascular complications in patients with polycythemia are age greater than 65 years old and previous history of thrombosis [5]. The patient in this report has a history of ischemic cerebrovascular disease at 2 years of age, heart disease, and polycythemia secondary to a Glenn procedure. The incidence of recurrent thrombotic events in a retrospective cohort of 1638 patients with polycythemia vera was 5% in patients under 65 years of age and 10.9% in those older than 65 years [6]. De Stefano et al. found an incidence of recurrent thrombosis in patients with polycythemia of 5.6% patients/year, where 60.8% were arterial and 39.7% were venous [5]. Other risk factors associated with retinal artery occlusive events especially in the pediatric population include hyperhomocysteinemia, which is an independent risk factor for cerebral and ocular vascular events [7]; all thrombophilia screening was negative. Manayath et al. reported a case of idiopathic branch retinal artery occlusion in an 8-year-old with negative workup that responded extremely well to intravenous methylprednisolone, and this has now been advocated as an useful treatment in idiopathic retinal artery occlusion in which an inflammatory process is the likely culprit [2]. Since our patient had a history of cardiovascular malformation and polycythemia, it is hard to determine the origin of the embolic process that caused the artery occlusion; however, we believe this patient developed transient hemiretinal artery occlusion secondary to the polycythemia caused by a partially corrected cyanotic heart disease [8]. The treatment algorithm for retinal artery occlusion, although well described, does not improve overall visual function because most patients present after the threshold for permanent retinal damage is established; the retina sustains irreversible damage when the central retinal artery has been obstructed for 90 to 100 minutes [9], with studies by Hayreh and Jonas describing gliosis formation and optic nerve atrophy by 240 minutes after obstruction [9]. At presentation, our patient had acute artery occlusion but apparently it reperfused partially. After normalization of the hematologic condition, an even better reperfusion was observed with recovery to useful vision of 20/200 at follow-up.

## 5. Conclusion

Retinal infarction in children is an extremely rare diagnosis, with very few cases reported in the literature. Cardiac malformations including pulmonary infundibular stenosis, mitral valve prolapse, aortic stenosis, and left ventricular hypertrophy are the most common causes of cardiac emboli; our patient had 2 of these factors. Regarding hypercoagulability, polycythemia and hyperhomocysteinemia are the most common causes. This patient also had an increased RBC count and hematocrit. Origin of the embolic event is hard to determine but apparently normalization in hematologic condition after phlebotomies resulted in improvement of retinal perfusion.

## Figures and Tables

**Figure 2:**